# Aspirin impairs acetyl-coenzyme A metabolism in redox-compromised yeast cells

**DOI:** 10.1038/s41598-019-39489-4

**Published:** 2019-04-16

**Authors:** Gianluca Farrugia, Maria Azzopardi, Christian Saliba, Godfrey Grech, Angelina S. Gross, Jelena Pistolic, Vladimir Benes, Neville Vassallo, Joseph Borg, Frank Madeo, Tobias Eisenberg, Rena Balzan

**Affiliations:** 1 Centre for Molecular Medicine and Biobanking, University of Malta, Msida, Malta; 2 Department of Physiology & Biochemistry, University of Malta, Msida, Malta; 3 Department of Pathology, University of Malta, Msida, Malta; 4 Institute of Molecular Biosciences, NAWI Graz, University of Graz, Graz, Austria; 5 Genomics Core Facility, European Molecular Biology Laboratory, Heidelberg, Germany; 6 Department of Applied Biomedical Science, University of Malta, Msida, Malta; 7 BioTechMed Graz, Graz, Austria; 8 Central Lab Gracia, NAWI Graz, University of Graz, Graz, Austria

## Abstract

Aspirin is a widely used anti-inflammatory and antithrombotic drug also known in recent years for its promising chemopreventive antineoplastic properties, thought to be mediated in part by its ability to induce apoptotic cell death. However, the full range of mechanisms underlying aspirin’s cancer-preventive properties is still elusive. In this study, we observed that aspirin impaired both the synthesis and transport of acetyl-coenzyme A (acetyl-CoA) into the mitochondria of manganese superoxide dismutase (MnSOD)-deficient *Saccharomyces cerevisiae* EG110 yeast cells, but not of the wild-type cells, grown aerobically in ethanol medium. This occurred at both the gene level, as indicated by microarray and qRT-PCR analyses, and at the protein level as indicated by enzyme assays. These results show that in redox-compromised MnSOD-deficient yeast cells, but not in wild-type cells, aspirin starves the mitochondria of acetyl-CoA and likely causes energy failure linked to mitochondrial damage, resulting in cell death. Since acetyl-CoA is one of the least-studied targets of aspirin in terms of the latter’s propensity to prevent cancer, this work may provide further mechanistic insight into aspirin’s chemopreventive behavior with respect to early stage cancer cells, which tend to have downregulated MnSOD and are also redox-compromised.

## Introduction

Aspirin (acetylsalicylic acid, ASA) is a widely used anti-inflammatory, cardioprotective and antithrombotic drug that has, in recent years, been found to have promising chemopreventive, antineoplastic properties^1–3^. These are attributed not only to aspirin’s anti-inflammatory and anti-platelet effects, but also to its propensity to induce programmed cell death, such as apoptosis, in cancer cells^2,4,5^. The full range of mechanisms underlying aspirin-induced apoptosis of malignant cells is still not fully understood, and has aroused considerable research efforts involving the use of several eukaryotic experimental models. One such model is the yeast *Saccharomyces cerevisiae*, long used to study apoptosis in living organisms since it retains several core eukaryotic cellular processes, including the hallmark features of apoptosis^6–9^. In particular, yeast serves as a valuable tool to study mitochondria-associated regulated cell death^10–12^.

Since mitochondrial manganese superoxide dismutase (MnSOD) deficiency can reprogram cells to a cancer prone form of metabolism (Warburg effect)^13^, we decided to use the redox-compromised, MnSOD-deficient *S*. *cerevisiae* EG110 cells as a model of cancer cells. In fact, cancer cells tend to manifest downregulated MnSOD during the early stages of their development^14–16^ and are also redox-compromised^17^.

In our previous studies, aspirin reproducibly and robustly induced an apoptotic phenotype in MnSOD-deficient *S*. *cerevisiae* cells, but not in wild-type yeast cells, during aerobic growth in ethanol medium^18–21^. We also showed that the aspirin-induced apoptosis in redox-compromised MnSOD-deficient yeast cells is associated with severe mitochondrial dysfunction, including events such as overproduction of mitochondrial superoxide radicals (O_2_˙^−^), oxidation of mitochondrial NAD(P)H^21^, decreased respiration, release of cytochrome *c* and disruption of the mitochondrial membrane potential^20^. Overall, these results indicated that the mitochondrial milieu constitutes a critical cellular target of aspirin.

In this study, we examined the effect of aspirin on the expression of genes involved in acetyl-coenzyme A (acetyl-CoA) generation and its transport into the mitochondria of MnSOD-deficient yeast cells growing aerobically on ethanol medium. Although acetyl-CoA is a metabolite of fundamental importance in eukaryotic organisms, since it drives the tricarboxylic acid (TCA) cycle required for energy generation, it remains one of the most poorly studied potential targets of aspirin in the context of the latter’s antineoplastic effects^2^. Hence, this work provides novel mechanistic insight into the metabolic changes associated with aspirin-mediated apoptosis. This may be of relevance to aspirin’s chemopreventive behavior in redox-compromised cancer cells during their early stages of development.

## Methods

### Yeast strains and plasmids

The yeast strains used in this study include the wild-type *Saccharomyces cerevisiae* EG103 (*MATα leu2-3 112 his3Δ1 trp1-289a ura3-52 GAL*^+^) and the MnSOD-deficient yeast strain EG110 (*EG103 sod2 Δ:: TRP1*), kindly provided by Edith Gralla, University of California, Los Angeles and Valeria C. Culotta, Johns Hopkins University, Baltimore.

### *ADH2*-overexpression in yeast

To generate *ADH2*-overexpressing strains (EG103 *pGPD-ADH2* and EG110 *pGPD-ADH2*) the constitutive glyceraldehyde-3-phosphate dehydrogenase promoter (pGPD) was introduced to the endogenous *ADH2* locus according to the method described by Janke *et al*.^22^. The plasmid pYM-N15 served as the template to generate a linear PCR fragment with primers 5′-ACAAAAAGCATACAATCAACTATCAACTATTAACTATATCGTAATACACAATGCGTACGCTGCAGGTCGAC-3′(ADH2_S1) and 5′-TTGCCGTTGGATTCGTAGAAGATAATGGCTTTTTGAGTTTCTGGAATAGACATCGATGAATTCTCTGTCG-3′(ADH2_S4 primer). Strains carrying an HA-tagged version of Adh2 were generated by similar approaches using pYM-N16 (EG103 *pGPD-3HA-ADH2* and EG110 *pGPD-3HA-ADH2*) or pYM-N14 (EG103 *ADH2-6HA* and EG110 *ADH2-6HA*) as templates and primers ADH2_S1 and ADH2_S4 or 5′-GGCATACTTGATAATGAAAACTATAAATCGTAAAGACATAAGAGATCCGCTTAATCGATGAATTCGAGCTCG-3′ (ADH2_S2) and 5′-AGATGGAGAAGGGCCAAATTGCTGGTAGATACGTTGTTGACACTTCTAAACGTACGCTGCAGGTCGAC-3′(ADH2_S3), respectively. Correct integration of linear DNA fragments was verified by colony PCR using primers 5′-CCTGTGTAACTGATTAATCCTGC-3′ (ADH2_up) and 5′-GTCGACCTGCAGCGTACG-3′ (S1/S3_reverse) for all N-terminal integrations and primers 5′-GGAAGAATTGTTTACCTCGCTC-3′ (ADH2 -fw) and S1/S3_reverse for C-terminal tagging.

### Culture conditions

Yeast cells were aerobically cultivated in YPE medium (1% (w/v) yeast extract, 2% (w/v) bactopeptone and 3% (v/v) ethanol) in the absence and presence of 15 mM aspirin (acetylsalicylic acid, ASA) (Sigma-Aldrich), at 28 °C with constant shaking at 250 rpm. The pH of the aspirin-treated YPE medium was adjusted to 5.5 using 1 M Trizma base (Sigma-Aldrich), prior to inoculation. For plates, cells were cultivated on YPD medium (1% (w/v) yeast extract, 2% (w/v) bactopeptone and 2% (w/v) glucose) containing 2% (w/v) agar, at a sustained incubation temperature of 28 °C.

### Measurement of cell growth and viability

Cell growth was measured as the optical density at 600 nm (OD_600_). Cell viability was measured by plating serial dilutions of yeast cell cultures onto YPD plates (500 cells per plate). The plates were left to incubate for at least 48 h at 28 °C after which colony forming units (cfus) were counted. The cfu counts of aspirin-treated cells were then presented as fold changes in viability by normalizing them to the cfu counts of untreated cells.

### RNA preparation for microarrays

Yeast cells were aerobically cultivated for 48 h (mid-log phase) in YPE medium in both the absence and presence of 15 mM ASA, at a temperature of 28 °C with constant shaking at 250 rpm. Harvested yeast cells were mechanically lysed by beadmilling, using a micro-minibeadbeater (Biospec), in the presence of acid-washed glass beads (500 nm diameter) and lysis buffer (RLT) provided by the Qiagen RNeasy Mini Kit. Three cycles of 60 s beadmilling followed by 60 s of cooling on ice were carried out. Total yeast RNA was extracted and purified using the Qiagen RNeasy Mini Kit, according to the manufacturer’s instructions. The quantity and quality of total RNA was determined using a Thermo Scientific Nanodrop spectrophotometer and an Agilent Bioanalyzer. Moreover, the RNA samples were subjected to reverse transcription polymerase chain reaction (RT-PCR) in order to verify that they were suitable for complementary DNA (cDNA) synthesis and not significantly contaminated by guanidine salts or ethanol, which interfere with the downstream microarray hybridization. One microgram of the RNA was used to synthesize cDNA using a Quantitect Reverse Transcription Kit (Qiagen). The cDNA was probed for the constitutive, intron-spanning *YRA1* gene using the custom-designed primers 5′-TGTCGGTGGTACTCGTGGTA-3′ (*YRA1*-F) and 5′-TAGTCCGCCATTTCCTTGTC-3′ (*YRA1*-R), at an annealing temperature of 54 °C, together with the Thermo Scientific 2X ReddyMix PCR Master Mix. The RT-PCR products were then visualized by electrophoresis on a 2% (v/v) agarose gel under UV light.

### Microarray hybridization and data analysis

Preparation of complimentary RNA and subsequent hybridization to whole yeast genome microarrays were carried out using the GeneChip Yeast Genome 2.0 Array (Affymetrix). Normalization of the resulting microarray data was carried out using the robust multi-array average (RMA) method^23^, implemented with AltAnalyze Version 2.0.8.1 (University of Cincinnati).

Normalized log2-expression values, fold changes, moderated *t*-statistics and corresponding *P*-values were determined for the probe sets of every individual *S*. *cerevisiae* gene present on the microarray chip. Differentially expressed genes with Benjamini-Hochberg-adjusted *P*-values < 0.05 and fold changes ≥1.5 (in presence of ASA *versus* absence of ASA) were identified. Microarray hybridization and analysis were carried out on three biological replicates.

The normalized microarray data sets were used for principle components analysis (PCA) to identify correlations and outliers among individual aspirin-treated and untreated samples. The normalized datasets were further analyzed by hierarchical cluster analysis. Both PCA and hierarchical clustering were carried out using the online ClustVis web tool^24^.

### Gene Ontology (GO) Analysis

Identification of enriched biological process, molecular function and cellular component GO categories was carried out by over-representation analysis (ORA) using GO-Elite software Version 1.2.6, which is integrated with AltAnalyze. Statistical significance of GO enrichment was determined using the Fisher Exact Test with a threshold *P*-value < 0.05.

All microarray datasets related to this study were deposited in the GEO (NCBI) repository and are accessible through the Accession number: GSE115660.

### Microarray result validation by quantitative polymerase chain reaction (qRT-PCR)

The respective messenger RNA (mRNA) levels of differentially expressed *S*. *cerevisiae* target genes were determined by qRT-PCR to validate the microarray results. The extraction of total RNA from yeast cells, verification of its quality and the subsequent preparation of cDNA were carried out as described in a previous section. Each sample of resulting cDNA was probed in triplicate for target genes using the custom-designed oligonucleotide primer pairs (Integrated DNA Technologies) shown in Supplementary Table S1a. This was carried out by using the 2X Quantitect SYBR-Green PCR Master Mix (Qiagen) and Rotor Gene Q Thermocycler (Qiagen), according to the manufacturer’s instructions. Target gene expression in the samples was normalized against the geometric average expression of *GLC7* and *SMD2* reference genes as an internal control (Supplementary Table S1b). These reference genes exhibit constitutive expression which does not change significantly across the different experimental conditions of this study, including the absence and presence of ASA (Supplementary Table S2). The calculation, based on the ∆∆C_*t*_ method, of normalized relative quantities (NRQs) of target genes as a measure of their differential expression in yeast cells grown in the absence and presence of aspirin, was carried out using qbase + (Biogazelle). Differentially expressed genes with significant *P*-values < 0.05 and fold changes >1.5 (in presence of ASA *versus* absence of ASA) were identified. The qRT-PCR assays were carried out on three biological replicates.

### Enzyme activity measurements

Yeast total cell extracts, cultivated for 48 h in ethanol medium in the absence and presence of aspirin, were prepared by beadmilling, using a micro-minibeadbeater (Biospec), in the presence of acid-washed glass beads (500 nm diameter). Alcohol dehydrogenase (ADH) activity was measured according to Vallee and Hoche^25^, acetyl-coenzyme A synthetase (ACS) activity was measured according to van den Berg *et al*.^26^ and carnitine acetyl transferase (CAT) enzyme activity was quantified according to Chase^27^. Peroxisomal citrate synthase activity was measured according to Srere^28^ in yeast cytosolic fractions previously isolated from the mitochondria according to Glick and Pon^29^. The protein concentrations of total and cytosolic yeast cell extracts were determined with the Pierce Bicinchoninic Acid Assay Kit (Pierce), using bovine serum albumin (BSA) as a protein standard.

### Immunoblotting

Immunoblotting after sodium dodecyl sulfate polyacrylamide gel electrophoresis (SDS-PAGE) of the cytosolic fractions to confirm the absence of any mitochondrial protein, was carried out using standard protocols, with antibodies specific for yeast cytosolic glucose-6-phosphate dehydrogenase (anti-G6PD, Sigma-Aldrich, A9521, 1:7500) and mitochondrial heat shock protein 60 (anti-Hsp60, Abcam, ab59458, 1:1000).

Immunoblotting after SDS-PAGE of chemically lysed yeast^30^ was performed using standard protocols with antibodies specific for yeast glyceraldehyde-3-phosphate dehydrogenase (anti-GAPDH, ThermoFisher, MA5-15738, 1:10.000) and HA (Sigma, H9658, 1:10000).

### Statistical analyses

Pairwise comparisons between treatments and controls were examined using the unpaired, two-tailed *t*-test (GraphPad Prism, Version 6.0). Multiple comparisons between treatments and controls were analyzed by one-way ANOVA with the post-hoc Bonferroni test. Where appropriate, the nonparametric Mann-Whitney U-test and Kruskal-Wallis test were applied to confirm the results of the *t*-tests and one-way ANOVA tests, respectively. Significance was accepted at a *P*-value < 0.05.

## Results

### Aspirin induces differential expression of genes involved in mitochondrial acetyl-CoA metabolism and transport

**Figure 1:**
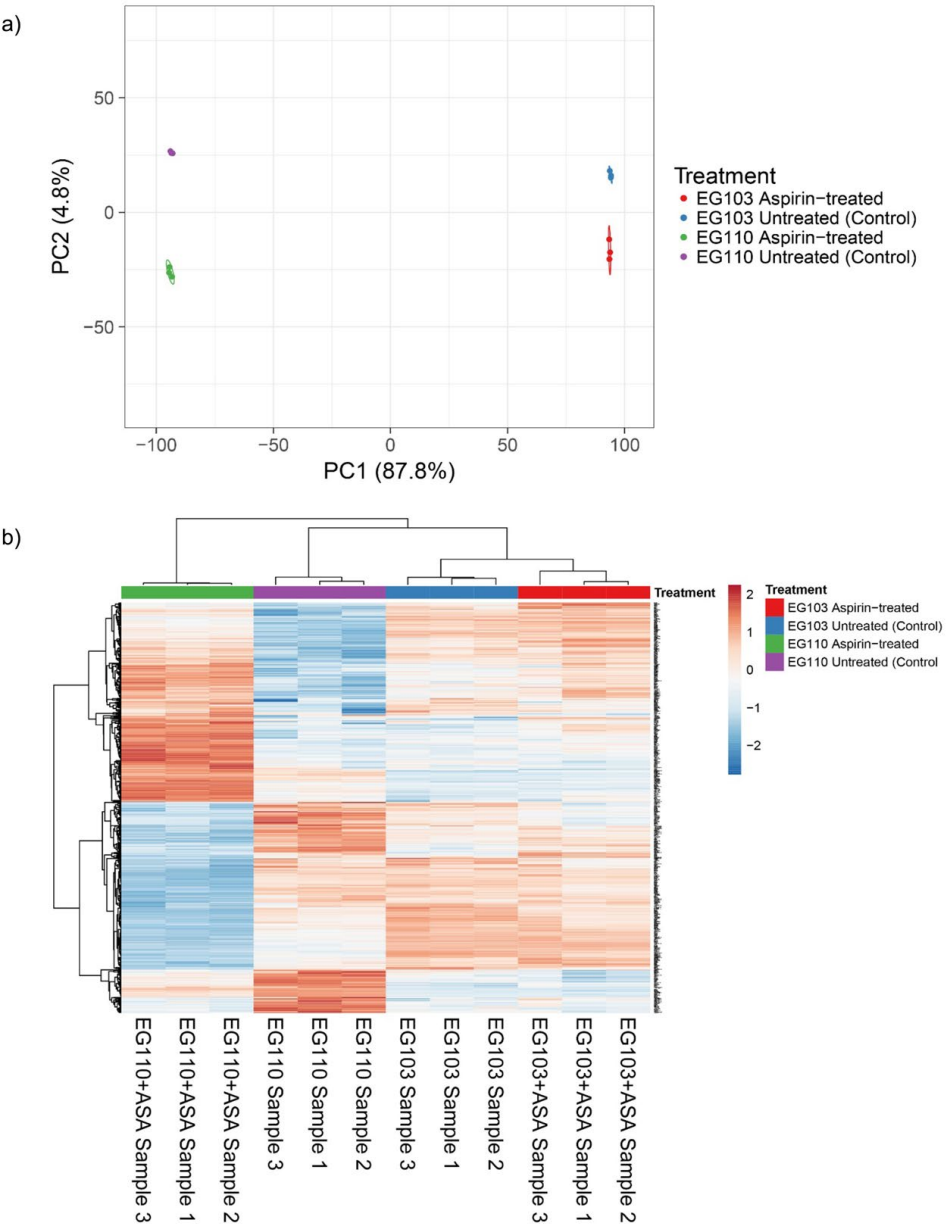
Principal components analysis (PCA) and heatmaps of normalized expression data obtained from redox-compromised EG110 yeast cells and wild-type EG103 yeast cells grown in ethanol medium in the absence and presence of aspirin. (**a**) Principle Component Analysis (PCA) two dimensional scatter plot of normalized expression data obtained from aspirin-treated (green and red dots) and untreated (control – purple and blue dots) samples of redox-compromised, MnSOD-deficient EG110 and wild-type EG103 yeast cells, respectively, cultivated for 48 h in ethanol medium. Three biological replicates of each were considered. Axis X = PC1: PCA Component 1 (87.8% variance) separating EG110 from EG103 yeast cells; Y = PC2: PCA Component 2 (4.8% variance) separating aspirin-treated cells from untreated cells. (**b**) Heatmap depicting differential gene expression due to aspirin in individual replicate samples of EG110 and EG103 yeast cells, as compared to untreated EG110 and EG103 yeast cells after 48 h of cultivation in ethanol medium. The heat map shows all genes that were significantly changed by aspirin in EG110 cells. The expression of these same genes was also followed in the wild-type EG103 cells, in the absence and presence of aspirin. Columns represent biological replicates, whereas rows represent genes. Gene expression levels are shown using a pseudocolour scale (−2.0 to 2.0) with red denoting high expression levels and blue denoting low expression levels.

The mechanism of aspirin-induced mitochondrial deterioration is not fully understood. To further characterize changes upon aspirin insults, we performed a transcriptome analysis of MnSOD-deficient (EG110) and MnSOD-proficient (EG103) yeast cells grown in ethanol medium. Principle Components Analysis (PCA) (Fig. 1a) of the normalized EG110 and EG103 microarray datasets indicated tight clustering and distinct separation of redox-compromised EG110 biological replicates from wild-type EG103 biological replicates, with 87.8% of the variance mainly explained by principle component 1 (PC1). Likewise, distinct clustering of aspirin-treated *versu*s untreated EG110 sample groups was observed along principal component 2 (PC2), with a variance of 4.8%. This distinct clustering of aspirin-treated *versu*s untreated EG110 sample groups was corroborated by the hierarchical clustering heatmaps (Fig. 1b), showing that aspirin clearly induced a strong and uniform transcriptional change in mRNA expression of MnSOD-deficient EG110 replicates.

**Figure 2:**
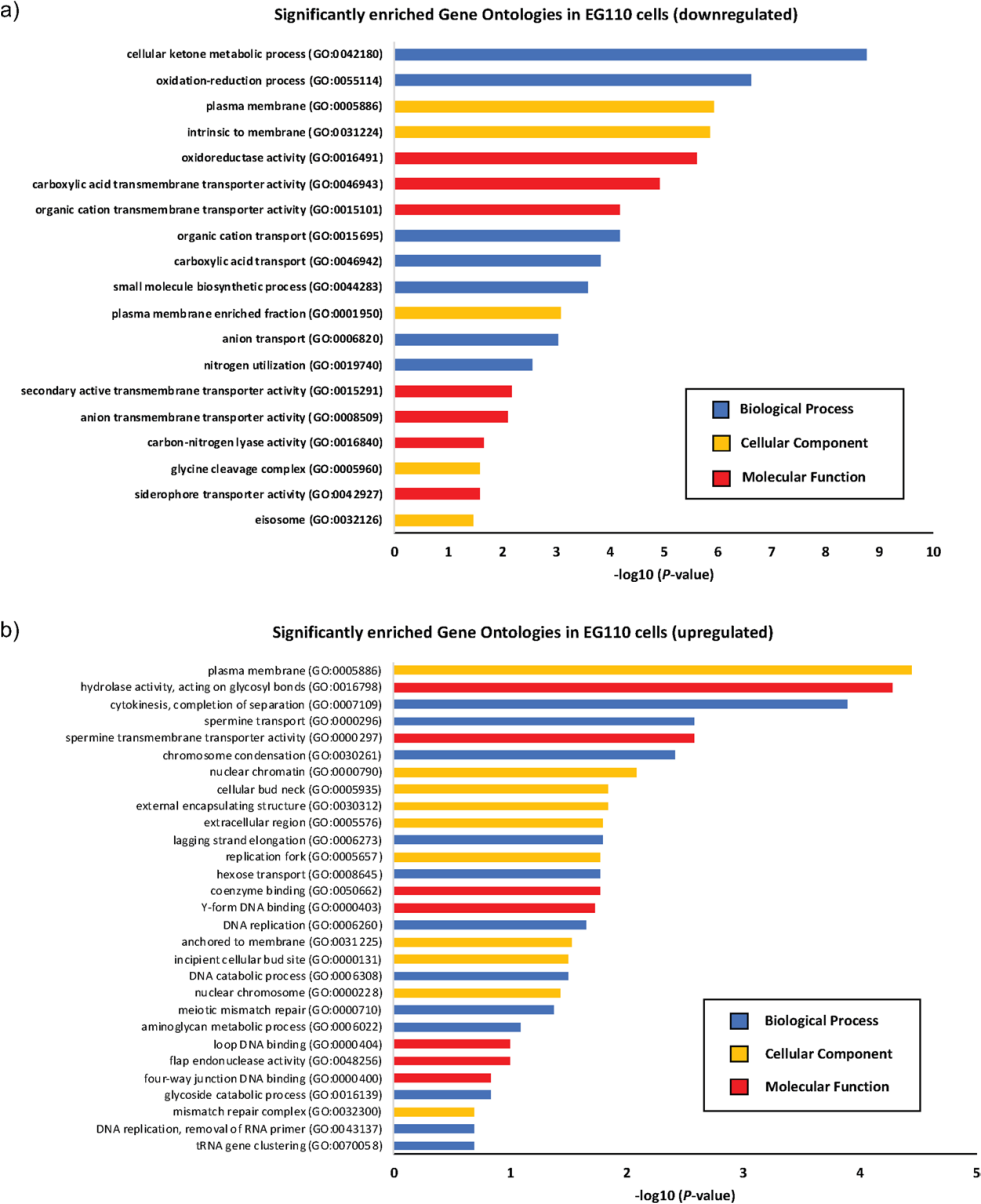
Gene Ontology (GO) analysis of differentially expressed mRNA transcripts in aspirin (ASA)-treated MnSOD-deficient EG110 yeast cells. All statistically significant GO terms of downregulated (**a**) and upregulated (**b**) mRNA transcripts (>1.5-fold) in ASA-treated EG110 are shown, each belonging to one of the following GO categories: biological processes (blue bars), cellular components (yellow bars) or molecular function (red bars). The calculated -log10 (*P*-value) values reflect the statistical significance of each GO term enrichment. The genes selected for further study, which include *ACS1*, *ADH2*, *CIT2*, *CAT2*, *YAT2* and *AGP2* are associated with the enriched cellular ketone metabolic process ontology.

**Figure 3:**
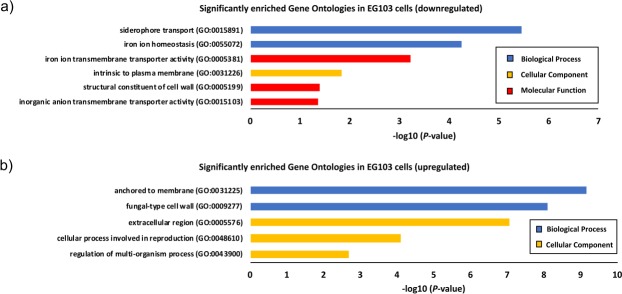
Gene Ontology (GO) analysis of differentially expressed mRNA transcripts in aspirin (ASA)-treated wild-type EG103 yeast cells. All statistically significant GO terms of downregulated (**a**) and upregulated (**b**) mRNA transcripts in ASA-treated EG103 cells are shown, each belonging to one of the following GO categories: biological processes (blue bars), cellular components (yellow bars) or molecular function (red bars). The calculated −log10 (*P*-value) values reflect the statistical significance of each GO term enrichment.

The normalized, wild-type EG103 yeast microarray datasets also indicated distinct clustering and separation of aspirin-treated *versus* untreated biological replicates, along PC2 (Fig. 1a), corroborated by the accompanying heatmaps shown in Fig. 1b, which demonstrate that aspirin likewise altered mRNA transcription in EG103 yeast cells, but to a much lesser extent than in EG110 cells. In fact, follow-up application of Gene Ontology (GO) analysis for MnSOD-deficient EG110 cells (Fig. 2) and EG103 cells (Fig. 3) suggests that aspirin had a relatively much lower effect on the wild-type EG103 cells in comparison to their redox-compromised EG110 counterparts, given that relatively few GO categories were enriched by aspirin in the wild-type strain (Fig. 3).

On the other hand, GO-analysis results obtained in MnSOD-deficient EG110 cells, additionally showed that aspirin significantly downregulated genes associated with at least 19 GO categories, the most strongly and significantly enriched being the ‘cellular ketone metabolic process’ (GO:0042180) ontology (Fig. 2), a category that did not appear enriched by aspirin in wild-type EG103 cells (Fig. 3). Aspirin-induced apoptosis involves mitochondria and mitochondrial metabolism-dependent processes^18,20,21,31,32^. In light of this, we selected a number of significantly downregulated genes associated with the enriched GO:0042180 category and involved in mitochondrial metabolism and metabolite transport (*ADH2*, *ACS1*, *CAT2*, *YAT2*, *CRC1*, *CIT2*, *SFC1* and *AGP2 -* Fig. 2 and Table 1), to further study their role in aspirin-induced cell death. The microarrays indicated that these selected genes were significantly downregulated by aspirin in redox-compromised MnSOD-deficient EG110 cells, but not in wild-type EG103 cells, after 48 h of growth in ethanol medium (see Table 1). Acetyl-CoA is a metabolite of central importance in eukaryotic organisms such as respiring yeast, since it drives the TCA cycle in mitochondria to facilitate energy generation.

**Table 1:** Aspirin (ASA)-induced differential expression of genes involved in acetyl-CoA synthesis and transport into the mitochondria of *Saccharomyces cerevisiae* EG110 and EG103 yeast cells grown in ethanol medium.

Affymetrix Probe Set	Ensembl ID	Gene Name	Encoded Protein	Fold Change		
				EG110 + ASA *vs* EG110	EG103 + ASA *vs* EG103	EG110 *vs* EG103^#^
1774070_at	YMR303C	*ADH2*	Ethanol-induced alcohol dehydrogenase isoform 2	−3.01***	−1.06^NS^	1.40**
1772915_at	YAL054C	*ACS1*	Acetyl-CoA synthetase isoform 1 (repressed by glucose)	−2.72***	1.01^NS^	1.08^NS^
1774377_at	YML042W	*CAT2*	Carnitine acetyltransferase (peroxisomal and mitochondrial)	−2.56***	1.04^NS^	−1.72***
1771757_at	YER024W	*YAT2*	Carnitine acetyltransferase (cytosolic)	−2.68***	1.05^NS^	−2.69***
1774778_at	YOR100C	*CRC1*	Mitochondrial inner membrane carnitine transporter	−4.52***	1.04^NS^	−1.62***
1773515_at	YCR005C	*CIT2*	Peroxisomal citrate synthase	−1.72***	1.01^NS^	−1.23*
1774072_at	YJR095W	*SFC1*	Mitochondrial succinate-fumarate transporter	−1.60***	−1.05^NS^	−1.01^NS^
1773859_at	YBR132C	*AGP2*	Plasma membrane carnitine transporter	−1.91***	−1.01^NS^	−1.80***

EG103 is the wild-type strain, whilst EG110 is the MnSOD-deficient yeast strain. Cells were cultivated in rich medium containing the non-fermentable carbon source ethanol (YPE) in the absence and presence of 15 mM aspirin (previously added to the medium with adjustment of the pH to 5.5 with 1 M Trizma base) for 48 h at 28 °C, 250 rpm. Microarray analysis of total yeast RNA to assess aspirin-induced differential gene expression was carried out using the GeneChip Yeast Genome 2.0 Array (Affymetrix), as described in Methods. The minus sign (−) indicates downregulation. ***(*P* < 0.001); **(*P* < 0.01); *(*P* < 0.05); NS, (*P* ≥ 0.05); aspirin treatment *vs* no treatment, ^#^untreated EG110 *vs* untreated EG103, unpaired two-tailed *t*-test.

Amongst the genes most significantly downregulated by aspirin in EG110 yeast cells, is the *ACS1* gene encoding one of 2 isoforms of acetyl-CoA synthetases (Acs1) (2.72-fold downregulation, *P* < 0.001; Table 1). Acs1 is an important source of acetyl-CoA which relies on the upstream acetaldehyde-derived supply of acetate during yeast cell growth in ethanol medium^26,33,34^. Under these growth conditions, acetaldehyde is itself derived from the oxidation of ethanol, catalyzed by the Adh2 isoform of alcohol dehydrogenase, encoded by the gene *ADH2*^35,36^, which was also markedly downregulated by aspirin in EG110 (3.01-fold downregulation, *P* < 0.001), but not in EG103 (MnSOD-proficient wild-type) yeast cells (Table 1).

During growth in non-fermentable media such as ethanol, acetyl-CoA molecules formed by peroxisomal and cytosolic acetyl-CoA synthetases must be converted into acetyl unit derivatives and shuttled into the mitochondria *via* the glyoxylate cycle and/or the carnitine shuttle^37,38^ in order to drive the TCA cycle. The microarray results indicated that the genes *CIT2* and *SFC1*, encoding essential glyoxylate cycle pathway proteins^38–40^, and the genes *CAT2*, *YAT2* and *CRC1*, which encode critical carnitine shuttle proteins^41–43^, were significantly downregulated in aspirin-treated EG110 (>1.5-fold downregulation, *P* < 0.001), but not in EG103, yeast cells (Table 1). This was also the case for *AGP2*, a gene encoding a yeast cell plasma membrane carnitine transporter protein, which facilitates cellular import of carnitine from the surrounding growth medium^44^.

**Figure 4:**
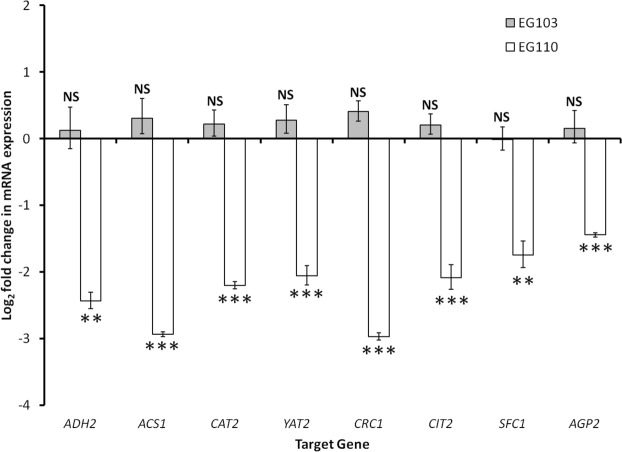
Aspirin-induced log_2_ fold change expression of target genes in wild-type (EG103) and MnSOD-deficient (EG110) *Saccharomyces cerevisiae* yeast cells grown in the absence and presence of aspirin (ASA). The figure shows aspirin-induced log_2_ fold change expression of the target genes *ADH2*, *ACS1*, *CAT2*, *YAT2*, *CRC1*, *CIT2*, *SFC1*, and *AGP2* in EG103 yeast cells (grey bars) and EG110 yeast cells (white bars), after 48 h of aerobic growth in YPE medium treated with 15 mM aspirin. Total RNA isolated from cultured yeast cells was analyzed by qRT-PCR using gene-specific primers (see Supplementary Table S1). In all cases, the expression of target genes was normalized against *GLC7* and *SMD2* reference genes using the comparative ∆∆C_t_ method in qbase + (Biogazelle). Results are the mean ± standard error of the mean (SEM), from three independent biological replicates. NS, not significant (*P* > 0.05); **, moderately significant (*P* < 0.01); ***, highly significant (*P* < 0.001); unpaired two-tailed *t*-test. *ADH2* – Alcohol Dehydrogenase 2, *ACS1* - Acetyl-CoA Synthetase 1; *CIT2* – CITrate synthase 2; *SFC1* – Succinate-Fumarate Carrier 1; *CAT2*, *YAT2* – Carnitine AcetylTransferases, *CRC1* – Carnitine Carrier 1, *AGP2* – high-Affinity Glutamine Permease 2, *GLC7* – GlyCogen 7, *SMD2* - Core Sm protein Sm D2.

The aspirin-induced differential expression of the aforementioned genes, as indicated by the microarrays, was validated by qRT-PCR analysis (Fig. 4) using primers listed in Supplementary Table S1.

Altogether, the microarray and qRT-PCR results strongly suggest that in MnSOD-deficient EG110 yeast cells grown in ethanol medium, aspirin significantly reduces transcription of several acetyl-CoA metabolism-associated genes, potentially disrupting the pathways involved in cytosolic acetyl-CoA synthesis and its transport into the mitochondria.

### Aspirin reduces the activities of key enzymes involved in acetyl-CoA synthesis and mitochondrial transport

Induced alterations in the expression profile of gene transcripts do not necessarily correlate with expression of their respective encoded proteins^45^. Moreover, post-translational modifications of encoded gene products are not taken into account by microarrays and qRT-PCR techniques. Hence, to verify the aspirin-induced impairment of acetyl-CoA metabolic pathways in EG110 yeast cells, as indicated by the microarray (Table 1) and qRT-PCR results (Fig. 4), the activities of enzymes encoded by the genes *ACS1*, *CAT2*, *YAT2*, *CIT2* and *ADH2* were measured in protein extracts of MnSOD-deficient EG110 and wild-type EG103 yeast cells grown for 48 h in ethanol medium, in the presence and absence of aspirin. These enzymes were selected for further investigation, using UV-visible spectrophotometric enzyme activity assays, on the basis of their importance in mediating acetyl-CoA synthesis and transport into the mitochondria.

In agreement with the microarray and qRT-PCR results, aspirin significantly decreased the overall acetyl-CoA synthetase enzyme activity in EG110, but not in EG103, yeast cells grown for 48 h in ethanol medium (Table 2). There are two known acetyl-CoA synthetase isoforms present in yeast cells - Acs1 and Acs2 (encoded respectively by *ACS1* and *ACS2*)^26,46^. Both of these isoforms actively ligate acetate to CoA to yield acetyl-CoA, however, the expression of Acs1 is known to be heavily de-repressed in the absence of glucose and in cases where there is a prevalence of non-fermentable carbon sources, such as ethanol and acetate^26,47^. Moreover, the affinity of Acs1 for acetate is reported to be several times greater than that of the constitutively expressed Acs2 isoform, suggesting that Acs1 is primarily responsible for acetyl-CoA synthesis during growth on non-fermentable carbon sources such as ethanol^26^. This implies that the aspirin-induced decrease of acetyl-CoA synthetase activity observed in redox-compromised EG110 yeast cells, largely reflects an impairment of Acs1 activity. This does not necessarily exclude an accompanying aspirin-induced impairment of Acs2 activity. However, the aspirin-induced decline in *ACS2* gene expression was less than 1.5-fold, as opposed to the 2.72-fold downregulation of *ACS1*.

**Table 2:** Enzyme activities in *Saccharomyces cerevisiae* cells grown for 48 h in ethanol medium in the absence and presence of aspirin (ASA)^b^.

Strains^a^	Specific enzyme activity (units mg^−1^ of total protein)			
	EG110		EG103	
	Control	+ASA	Control	+ASA
Alcohol dehydrogenase^c^	6.14 +/− 0.781	3.56 +/− 0.356**	14.72 +/− 1.869	13.99 +/− 1.016^NS^
Acetyl-CoA synthetase^d^	0.32 +/− 0.031	0.20 +/− 0.021**	0.46 +/− 0.017	0.53 +/− 0.047^NS^
Carnitine acetyltransferase^e^	0.20 +/− 0.005	0.14 +/− 0.005**	0.29 +/− 0.020	0.28 +/− 0.026^NS^
Peroxisomal citrate synthase^f^	0.37 +/− 0.044	0.17 +/− 0.038*	0.38 +/− 0.128	0.30 +/− 0.099^NS^

^a^EG103 is the wild-type strain, whilst EG110 is the MnSOD-deficient yeast strain.

^b^Cells were cultivated in rich medium containing the non-fermentable carbon source ethanol (YPE). Fifteen millimolar aspirin was added to the medium and the pH adjusted to 5.5 with 1 M Trizma base. Protein extracts were prepared after 48 h of cultivation. Acetyl-CoA synthetase, carnitine acetyltransferase, citrate synthase and alcohol dehydrogenase activity were measured as described in Methods. The values are the mean of at least three independent experiments (+/− standard error of the mean (SEM)).

^c^One unit of alcohol dehydrogenase is defined as the amount of enzyme forming 1 µmol of NADH per minute.

^d^One unit of acetyl-CoA synthetase is defined as the amount of enzyme forming 1 µmol of NADH per minute.

^e^One unit of carnitine acetyltransferase is defined as the amount of enzyme that catalyzes the acetylation of 1 µmol of reduced coenzyme A (CoASH) per minute.

^f^One unit of citrate synthase is defined as the amount of enzyme forming 1 µmol of reduced coenzyme A (CoASH) per minute.

***(*P* < 0.001); **(*P* < 0.01); *(*P* < 0.05); NS, (*P* ≥ 0.05); aspirin treatment *vs* no treatment, unpaired two-tailed *t*-test.

Similarly, aspirin decreased the overall carnitine acetyltransferase (CAT) enzyme activity in redox-compromised EG110, but not in wild-type EG103, yeast cells (Table 2). Yeast CAT enzymes include Cat2 present in both the mitochondria and peroxisomes, together with Yat2 present in the cytosol. These enzymes participate in the transport of acetyl-CoA-derived acetyl groups (in the form of acetylcarnitine) from the peroxisomes and cytosol to the mitochondria, reconverting them to acetyl-CoA upon entry into the mitochondria, to drive the TCA cycle^38,43,48^. Hence, the reduced activity of CAT enzymes observed in aspirin-treated EG110 yeast cells indicates an aspirin-induced impairment of the carnitine shuttle pathway in these cells during growth in ethanol medium, as initially suggested by the microarray and qRT-PCR results.

Likewise, the activity of peroxisomal citrate synthase (Cit2) declined significantly in MnSOD-deficient EG110 yeast cells, but remained unaltered in wild-type EG103 yeast cells cultivated for 48 h in aspirin-treated ethanol medium (Table 2). The activity of Cit2 was measured in cytosolic extracts that were free of mitochondrial protein, as verified by immunoscreening (Supplementary Fig. S1). This was important since yeast mitochondria contain the citrate synthases Cit1 and Cit3^49,50^, which would have interfered with the activity assay readings of peroxisomal Cit2 present in the cytosolic extracts.

The observed decrease of peroxisomal Cit2 activity in aspirin-treated EG110 yeast cells (Table 2) indicates that the glyoxylate cycle, which facilitates transport of acetyl-CoA-derived succinate (a TCA cycle intermediate) to the mitochondria^38^ is possibly impaired by aspirin much like the carnitine shuttle pathway.

Aspirin also caused a considerable decrease in Adh2 activity in MnSOD-deficient, redox-compromised EG110 yeast cells after 48 h of aerobic growth in ethanol medium, but not in wild-type EG103 cells (Table 2). This result corroborates the significant aspirin-induced downregulation of *ADH2* expression in EG110, but not in EG103, yeast cells observed in the microarray and qRT-PCR results.

### Increasing *ADH2* expression has no effect on growth and survival of aspirin-treated MnSOD-deficient yeast cells

In view of the pronounced aspirin-induced suppression of *ADH2* expression (3.01-fold downregulation, *P* < 0.001; Table 1) and Adh2 enzyme activity in MnSOD-deficient EG110 yeast cells (42% decreased activity, *P* < 0.01; Table 2), and taking into account the important upstream role of Adh2 in yeast acetyl-CoA synthesis during growth on ethanol medium^35,51,52^, we attempted to rescue the redox-compromised EG110 cells by increasing the expression of *ADH2* (and consequently of active Adh2). The *ADH2*-overexpressing strains EG110 *pGPD-ADH2*, and the respective wild-type EG103 *pGPD-ADH2* control, were constructed by replacing the endogenous promoter of *ADH2* with the constitutive *GPD* promoter.

**Figure 5:**
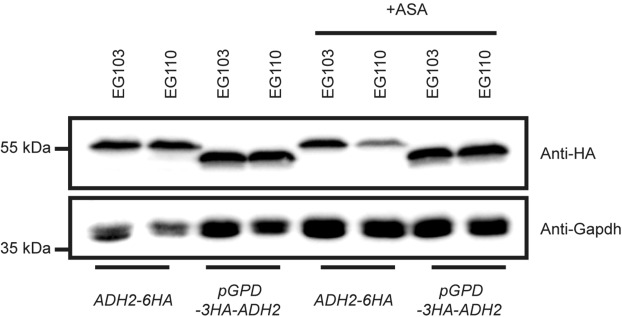
Representative immunoblot using a HA-specific antibody. EG103 is the wild-type strain, whilst EG110 is the MnSOD-deficient yeast strain. HA-tagged Adh2 (*ADH2-6HA* and *pGPD-3HA-ADH2*) strains were cultivated in YPE medium with or without ASA for 48 h and subjected to immunoblot analysis from whole cell extracts. The strains either carried the endogenous *ADH2* promotor or the constitutive *GPD* promotor (*pGPD*) as indicated. Full-length blots are presented in Supplementary Fig. S5.

Immunoblot analysis of strains carrying HA fusion constructs of the Adh2 protein allowed us to compare the quantity of expressed Adh2 after growth of the cells in ethanol medium, in the absence and presence of aspirin. This assay indicated an aspirin-induced decrease of Adh2 expression in MnSOD-deficient EG110 cells (Fig. 5). However, the immunoblots also showed that the amount of expressed Adh2 was greater in strains containing the *GPD* promoter.

**Figure 6:**
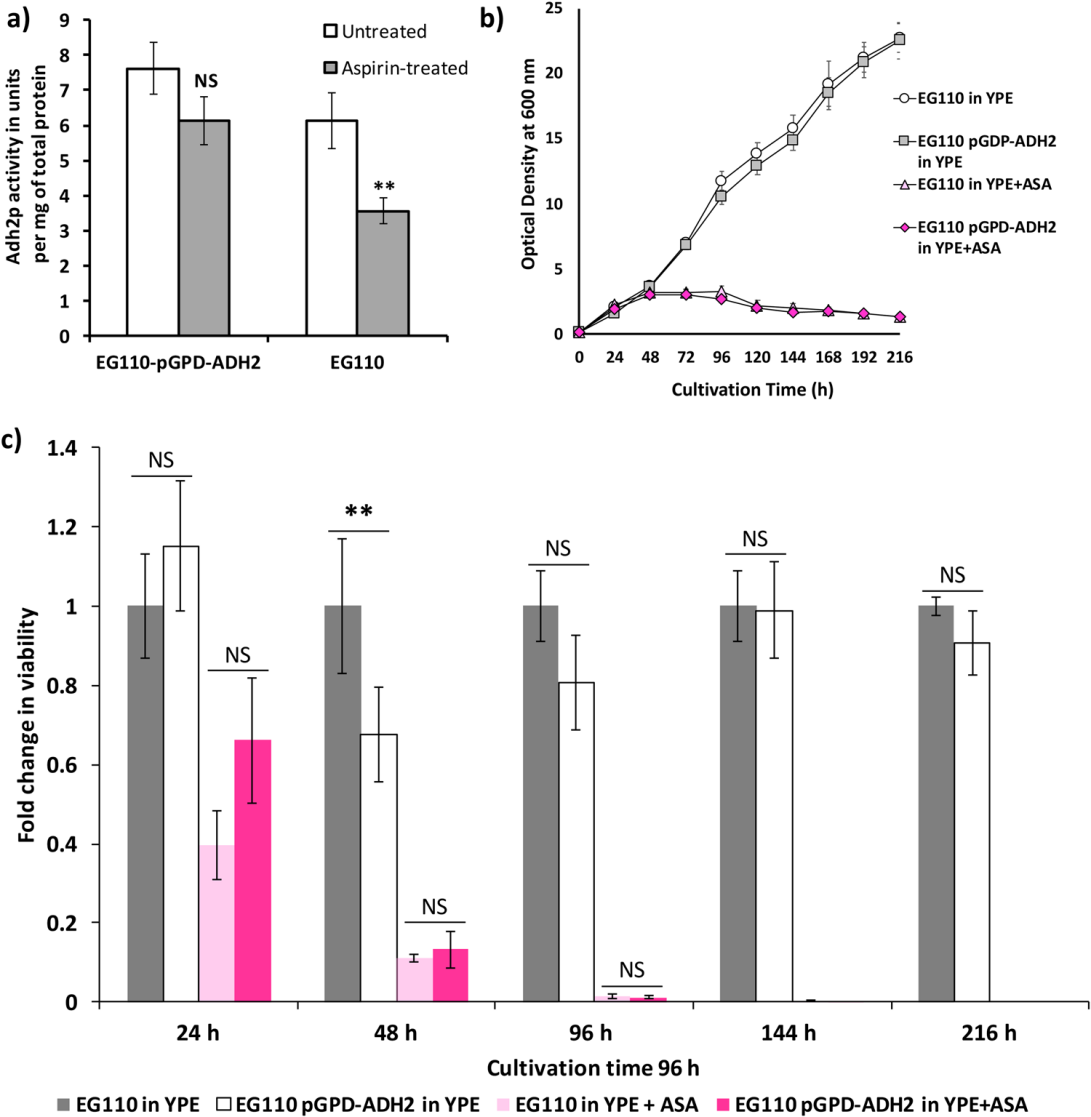
Comparison of (**a**) alcohol dehydrogenase (Adh2) activity, (**b**) growth and (**c**) viability of EG110-*pGPD-ADH2* to those of EG110 yeast cells, grown in ethanol medium (YPE) in the absence and presence of 15 mM aspirin (ASA). (**a**) Adh2 activity levels in total cell extracts of redox-compromised (MnSOD-deficient) *Saccharomyces cerevisiae* EG110 cells and recombinant EG110 *pGPD-ADH2* cells (EG110 cells where the endogenous *ADH2* gene promoter has been replaced with the yeast glyceraldehyde phosphate dehydrogenase (GPD) gene promoter) grown aerobically for 48 h in YPE medium at 28 °C, 250 rpm, in the absence (represented by the white bars) and presence (represented by the grey bars) of 15 mM ASA. Each vertical bar represents the mean of at least four experimental determinations of Adh2 activity. Error bars represent the standard error of the mean (SEM). NS, not significant (*P* > 0.05); **, moderately significant (*P* < 0.01); unpaired two-tailed *t*-test. (**b**) Growth curves of *S*. *cerevisiae* EG110 and EG110 *pGPD-ADH2* cells during aerobic cultivation in YPE medium in the absence and presence of 15 mM ASA at 28 °C (with constant shaking at 250 rpm). Each point represents the average of at least three independent sets of absorbance readings, measured at a wavelength of 600 nm. Error bars represent the SEM and appear where sufficiently large. (**c**) Fold change in viability of EG110 and EG110 *pGPD-ADH2* yeast cells grown in YPE medium in the absence and presence of aspirin, normalized against EG110 yeast cells grown in YPE medium, as determined from colony-forming unit (cfu) counts. Each vertical bar represents the mean of at least three independent determinations. Error bars represent the SEM. NS, not significant (*P* > 0.05); **, moderately significant (*P* < 0.01), (One-way ANOVA with Bonferroni post-hoc tests).

We next compared Adh2 activity of the Adh2-overexpressing strain to that of EG110 after 48 h of growth in aspirin-treated ethanol medium. As a result of the overexpression, EG110 *pGPD-ADH2* cells sustained a constant level of Adh2 activity even in the presence of aspirin, whereas the EG110 cells did not (Fig. 6a).

However, the sustained Adh2 enzyme activity did not confer any significant benefit, in terms of growth and survival, to EG110 *pGPD-ADH2* cells, either in the absence or presence of aspirin (Fig. 6b,c). EG110 *pGPD-ADH2* cells failed to show improved viability compared to EG110 yeast cells after treatment with aspirin (Fig. 6c).

Thus, restoring Adh2 activity alone was insufficient to prevent aspirin-induced toxicity in redox-compromised MnSOD-deficient yeast cells, even though Adh2-mediated ethanol oxidation into acetaldehyde lies upstream of acetyl-CoA synthesis^36^.

## Discussion

The principal scope of this investigation was to further understand the mechanisms underlying aspirin-induced apoptosis, which takes place in redox-compromised MnSOD-deficient *S*. *cerevisiae* EG110 yeast cells, but not in wild-type EG103 yeast cells, after cultivation in ethanol medium^18^. With this aim in mind, we first carried out microarray studies to investigate potential aspirin-induced differential gene expression in EG110 and EG103 yeast cells, after 48 h of cultivation (early to mid-log phase). The distinct response to aspirin of these two strains, described in past studies carried out in our laboratory^18–20^, was indeed again made immediately apparent in our transcriptome analysis, which showed that aspirin altered the gene expression profile of EG110 cells to a greater and significantly different extent than that of EG103 cells (Figs 1–3). This is particularly true for genes involved in acetyl-CoA synthesis and transport (Table 1).

The dissimilarity in the magnitude of aspirin’s effect on EG110 *versus* EG103 gene expression profiles can be readily explained by the deficiency of the mitochondrial antioxidant enzyme MnSOD in mutant EG110 (but not in wild-type EG103) cells that imparts very distinct gene expression profiles to these two strains (Fig. 1). In fact, GO analysis revealed several enriched pathways in aspirin-untreated EG110 cells with respect to EG103 yeast cells (Supplementary Fig. S4). Interestingly, overall altered expression of genes due to genotypic differences between the two strains accounted for the greatest variance between all analyzed conditions (Fig. 1a). One can speculate that some of these genotype-induced transcriptional adaptations are involved in unfolding the sensitivity to aspirin in MnSOD-deficient EG110 cells. For instance, genes involved in acetyl-CoA synthesis and transport were altered due to the genotype differences, irrespective of aspirin treatment. Several of these genes were already reduced due to the genotype and thus, aspirin further aggravated their down-regulation (Table 1).

**Figure 7:**
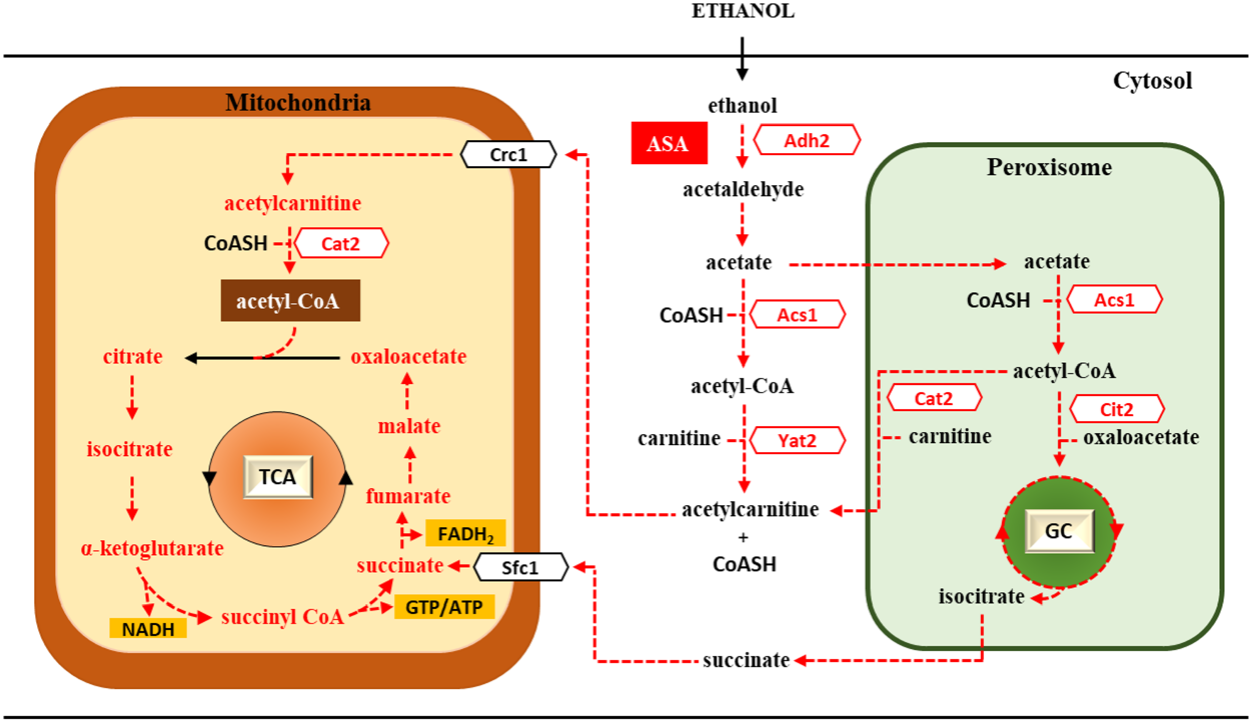
Aspirin-induced impairment of acetyl-CoA metabolism. Aspirin (ASA) downregulates genes involved in the synthesis (*ADH2* and *ACS1*) and transport (such as *CAT2*, *YAT2* and *CIT2*) of acetyl-CoA to the mitochondria and decreases the activity of their gene products in the MnSOD-deficient *Saccharomyces cerevisiae* EG110 cells (downregulated pathways shown in dotted red lines). ATP, adenosine triphosphate; CoA, coenzyme A; CoASH, reduced coenzyme A; FADH_2,_ reduced flavin adenine dinucleotide; GC, glyoxylate cycle; GTP, guanosine triphosphate; MnSOD, manganese superoxide dismutase; NADH, reduced nicotinamide adenine dinucleotide; TCA, tricarboxylic acid cycle.

The microarray results presented in Table 1 and validated by qRT-PCR (Fig. 4) notably indicated that, in EG110 cells grown in ethanol medium, aspirin significantly downregulated the expression of genes involved in central energy metabolism, particularly in pathways of acetyl-CoA synthesis and metabolite transport to the mitochondria. Subsequent enzyme activity assays confirmed that aspirin markedly impaired the activity of a number of key enzymes required for these acetyl-CoA pathways in MnSOD-deficient cells, but not in wild-type cells. Prominent among these impaired enzymes are the *ADH2*-encoded isoform of alcohol dehydrogenase, Adh2, and the yeast acetyl-CoA synthetase (ACS) enzymes (Table 2). As illustrated in Fig. 7, these enzymes play crucial successive roles in the synthesis of acetyl-CoA in yeast cells grown in ethanol medium^26,33–36^. Whilst this is the first reported incidence of aspirin-induced inactivation of Adh2 and ACS activity in yeast, aspirin has been shown to exert an inhibitory effect on gastric alcohol dehydrogenase activity in humans^53,54^. Furthermore, the aspirin metabolite, salicylate, has been reported to impair mammalian medium-chain acyl-CoA synthetase activity *in vitro*^55^.

Likewise, in EG110 cells, aspirin impaired the activities of yeast carnitine acetyltransferases and peroxisomal citrate synthase (Table 2), which respectively play indispensable roles in the carnitine shuttle and glyoxylate pathways to facilitate transport of acetyl-CoA to the mitochondria^37,38,43^ (Fig. 7). Similar findings in mammalian cell models have shown that salicylates interfere with carnitine shuttle transport, by inactivation of carnitine acetyl transferases^56^ and sequestration of both carnitine and reduced coenzyme-A^57^.

Our results, therefore, indicate that in redox-compromised EG110 yeast cells grown in ethanol medium, aspirin heavily interfered with the synthesis of acetyl-CoA and its supply to the mitochondria. This could well account for the energy failure and widespread mitochondrial dysfunction previously observed in aspirin-treated MnSOD-deficient yeast cells, as indicated by a decreased respiratory rate, disrupted mitochondrial membrane potential, and release of cytochrome *c*^20^. Interestingly, since acetyl-CoA is essential for the acetylation status of chromatin histones^58^, inhibition of acetyl-CoA synthesis as indicated in this study might partly explain the downregulation of our genes of interest in aspirin-treated EG110 yeast cells (Table 1, Fig. 4). In support of this, Takahashi and colleagues^59^ showed that inactivation of ACS enzymes is followed by rapid histone deacetylation and significant downregulation of gene expression in yeast. More recent studies have shown that salicylate caused profound histone deacetylation in human leukemia, osteosarcoma and colorectal cancer cell lines^60–62^.

The aspirin-induced disruption of both acetyl-CoA synthesis and transport in EG110 yeast cells, may also have occurred as a consequence of dysfunctional mitochondria, already rendered prone to oxidative stress by their lack of MnSOD. Past work carried out in our laboratory has consistently shown that aspirin-induced apoptosis of MnSOD-deficient yeast cells is preceded by mitochondrial dysfunction-associated events^20^. Prominent among these is the accumulation of mitochondrial superoxide radicals, the attenuation of which (*via* introduction of *Escherichia coli* iron-superoxide dismutase into the mitochondria of EG110 yeast cells) was a key factor in averting aspirin-induced apoptosis^21^. Moreover, in the present study, the induced overexpression of active Adh2 (Fig. 6a), which was heavily impaired by aspirin (Table 2) and lies upstream of all other acetyl-CoA synthesis and transport pathways in yeast grown on ethanol medium^36^ (Fig. 7), failed to prevent aspirin-induced cell death (Fig. 6b,c). This result might suggest that aspirin-induced impairment of yeast Adh2 (Table 2) is due to underlying mitochondrial dysfunction, since activity of this enzyme is regulated by the functional state of mitochondria and is positively induced by metabolites involved in the TCA cycle^63^. In light of all of this, it is strongly implied that the mitochondrial milieu plays a critical role in aspirin-mediated EG110 cell death. In fact, it is not surprising that perhaps, due to the pleiotropic effects of aspirin, restoring activity of a single enzyme within the acetyl-CoA synthesis pathway from ethanol, such as with the overexpression of active Adh2 (Fig. 6a), was not sufficient to completely overcome aspirin-induced mitochondrial acetyl-CoA deficiency and cell death.

The suggested influence of aspirin on acetyl-CoA metabolic pathways in our redox-compromised yeast cells may provide the key for understanding the chemopreventive, antineoplastic effects of aspirin in early developing tumour cells, which also suffer increased oxidative stress and altered metabolism^17,64–66^. The depletion of acetyl-CoA by aspirin would very likely exert an apoptotic effect on target cancer cells, since acetyl-CoA plays a central role in critical cellular pathways such as energy generation and biosynthesis. For example, acetyl-CoA serves as a critical precursor substrate for the *de novo* synthesis of fatty acids mediated by cytosolic fatty acid synthase (FASN), known to be a central factor underlying the survival, uncontrolled proliferation and progression of numerous types of cancer cells^67,68^. The importance of acetyl-CoA metabolism in cancer cells is further underscored by studies which show that the rate of synthesis of acetyl-CoA, mediated by the mammalian cytosolic enzyme acetyl-CoA synthetase (ACSS2), is significantly elevated in several malignant cell lines^67,68^ in order to satisfy their high energy requirements, particularly under conditions of metabolic stress.

The strong reliance of cancer cells on metabolic pathways that supply acetyl-CoA, has recently raised interest in the potential use of antineoplastic agents targeting acetyl-CoA. For instance, studies have shown that targeted inhibition of ACSS2-mediated acetyl-CoA synthesis caused growth inhibition of cultured tumor cells and significantly reduced the growth and size of tumor xenografts in mice^67^. Likewise, targeted disruption of key carnitine shuttle enzymes mediating transport of acetyl-CoA to the cytosol of lymphoma cancer cells for fatty acid synthesis, was shown to cause a cytotoxic depletion of acetyl-CoA and growth inhibition in these cells^69^. In addition, cross-talk between acetylation and histone modifications strongly implicate acetyl-CoA in the regulation of chromatin acetylation and gene expression^70,71^. Hence, the strong association between aspirin and impairment of acetyl-CoA metabolism, highlighted in this study, merits further investigation in the context of aspirin’s anti-cancer properties.

## Supplementary information

Farrugia et al., 2018 - Supplementary Information_ Aspirin impairs acetyl-coenzyme A metabolism in redox-compromised yeast cells
