# Supplementary material for: Aspirin impairs acetyl-coenzyme A metabolism in redox-compromised yeast cells
Source: Sci Rep. 2019 Apr 16;9:6152. doi: 10.1038/s41598-019-39489-4 (PMC6468118; doi:10.1038/s41598-019-39489-4)
Supplement: Supplementary file 1 — Farrugia et al., 2018 - Supplementary Information_ Aspirin impairs acetyl-coenzyme A metabolism in redox-compromised yeast cells [file 41598_2019_39489_MOESM1_ESM.pdf]

Farrugia *et al.*, 2018 - Supplementary Information

Aspirin impairs acetyl-coenzyme A metabolism in redox-compromised yeast cells

Gianluca Farrugia[1,2], Maria Azzopardi[1,2], Christian Saliba[1], Godfrey Grech[3], Angelina S. Gross[4], Jelena Pistolic[5], Vladimir Benes[5], Neville Vassallo[2], Joseph Borg[6], Frank Madeo[4, 7] Tobias Eisenberg[4, 7, 8] and Rena Balzan[1,2*]

[1] Centre for Molecular Medicine and Biobanking, University of Malta, Msida, Malta
[2] Department of Physiology & Biochemistry, University of Malta, Msida, Malta
[3] Department of Pathology, University of Malta, Msida, Malta
[4] Institute of Molecular Biosciences, NAWI Graz, University of Graz, Graz, Austria
[5] Genomics Core Facility, European Molecular Biology Laboratory, Heidelberg, Germany
[6] Department of Applied Biomedical Science, University of Malta, Msida, Malta
[7] BioTechMed Graz, Graz, Austria.
[8] Central Lab Gracia, NAWI Graz, University of Graz, Graz, Austria.
(*Corresponding author email address – rena.balzan@um.edu.mt)

Supplementary Information (SI) Guide

The file entitled 'Farrugia *et al*., 2018 - Supplementary Information' contains:

Supplementary Tables S1 showing qRT-PCR (a) target gene primers and (b) reference gene primers;

Supplementary Table S2 showing microarray fold change expression of candidate RT-PCR reference genes.

Supplementary Figures S1 to S3 and S5, which collectively show all our immunoblot results.

Supplementary Figure S4, which shows the GO analysis of differentially expressed mRNA transcripts in mutant, manganese superoxide-dismutase (MnSOD)-deficient, EG110 yeast cells with respect to wild-type, MnSOD-proficient, EG103 yeast cells.

References.

Supplementary Table S1a. **qPCR target gene primers used in this study**

Primer Name	Primer Sequence	Annealing Temperature (ºC)
ADH2 - Forward	5-TAGCGCAGTCGTTAAGGCTA-3'	54
ADH2 - Reverse	5'-GCAAACCAACCAAGACAACA-3'	
ACS1 - Forward	5'-ACGGGTTGCAGGACTATCAG-3'	54
ACS1 - Reverse	5'-CGTTTTAGGGTCTGGGATGA-3'	
AGP2 - Forward	5'-GCACGTCCAGTTGATTGCTA-3'	54
AGP2 - Reverse	5'-TTGGAACACACCAAAGAGCA-3'	
CAT2 - Forward	5'-CGTGCCCGAATTGAAGTCTA-3'	54
CAT2 - Reverse	5'-CATGTGCTCCGAGAAGTCCT-3'	
CIT2 - Forward	5'-TTTGGACCCAGAAGACGGTA-3'	54
CIT2 - Reverse	5'-GTTGGAACCTCGCCAGTTAG-3'	
CRC1 - Forward	5'-CCAATTCCGTAAAGGGGTTC-3'	54
CRC1 - Reverse	5'-TCGTTACTGCCTCCTTGCTT-3'	
SFC1 - Forward	5'-GGCAATCCGTTTCTCGTCT-3'	54
SFC1 - Reverse	5'-ACAACTTCCATCGGGTTCAC-3'	
YAT1 - Forward	5'-GGCTATTCGGCTCCAGTAGA-3'	54
YAT1 - Reverse	5'-CAGCACGTCGAACCAGTAGA-3'	
YAT2 - Forward	5'-GCTTTGCCAAACATCACTCA-3'	54
YAT2 - Reverse	5'-CAGAAATGGAGCCATTGACA-3'	

Supplementary Table S1b. **qPCR reference gene primers used in this study**

Primer Name	Primer Sequence	Annealing Temperature (ºC)
GLC7 - Forward	5'-GAGGATCTAAACCTGGTCAACAA-3'	54
GLC7 - Reverse	5'-TATTGCCCATGAATGTCACC-3'	
SMD2 - Forward	5'-TGGTGACAAGAACACCTGTGA-3'	54
SMD2 - Reverse	5'-GCCCTTCTTCTCTGTCCAAA-3'	

Supplementary Table S2. **GeneChip Yeast Genome 2.0 Microarray (Affymetrix) fold change (FC) expression of candidate reverse transcriptase polymerase chain reaction (RT-PCR) reference genes**

Probesets	Systematic Name Ensembl ID	Gene Symbol	Definition and Metabolic Function	EG110 + ASA vs EG110 FC	EG110 + ASA vs EG110 Log2 FC	Adjusted *P*-value	EG103 + ASA vs EG103 FC	EG103 + ASA vs EG103 Log2 FC	Adjusted *P*-value	Significance of FC
1777225_at	YER133W	***GLC7***	**Type 1 serine/threonine protein phosphatase catalytic subunit, involved in many processes (eg: glycogen metabolism, sporulation, mitosis); accumulates at mating projections by interaction with Afr1p; interacts with many regulatory subunits [Source:SGD;Acc:S000000935]**	-1.083	-0.116	0.244	-1.047	-0.066	0.599	NS
1770868_at	YLR275W	***SMD2***	**Core Sm protein Sm D2; part of heteroheptameric complex (with Smb1p, Smd1p, Smd3p, Sme1p, Smx3p, and Smx2p) that is part of the spliceosomal U1, U2, U4, and U5 snRNPs; homolog of human Sm D2 [Source:SGD;Acc:S000004265]**	-1.066	-0.092	0.346	1.108	0.148	0.297	NS

Not signficant (NS), $P > 0.05$, aspirin treatment versus no treatment, moderated t-test.

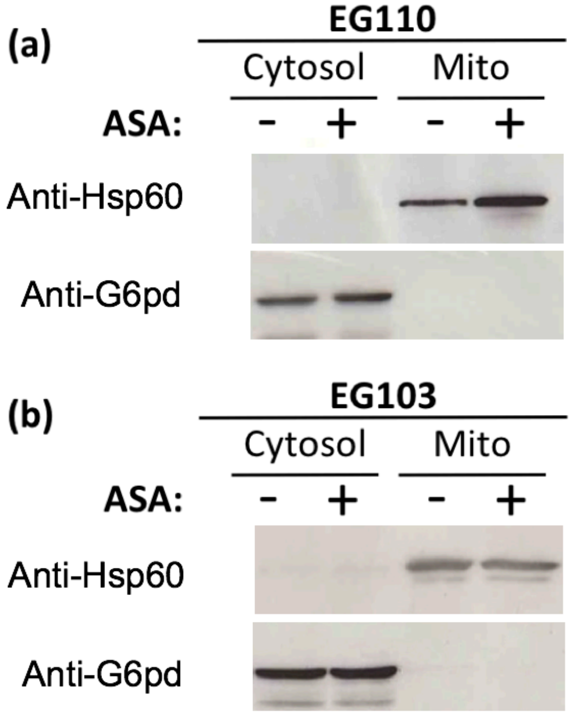

Supplementary Figure S1. Immunoscreening by chemiluminescence to confirm lack of cross-contamination between cytosolic and mitochondrial extracts. Immunoscreening by chemiluminescence for the cytosolic protein glucose-6-phosphate dehydrogenase (G6pd) (using polyclonal rabbit anti-G6pd[72], Sigma-Aldrich, A9521, 1:7500) and the mitochondrial matrix protein Hsp60 (using monoclonal mouse anti-Hsp60[73], Abcam, ab59458, 1:1000) was carried out in both the cytosolic and mitochondrial extracts of *Saccharomyces cerevisiae* (a) EG110 (MnSOD-deficient) and (b) EG103 (wild-type) cells grown aerobically in YPE medium in the absence (-) and presence (+) of aspirin (ASA). The electroblotting on to the nitrocellulose membranes was carried out from 5% (w/v) stacking, 12% (w/v) resolving acrylamide gels after sodium dodecyl sulfate polyacrylamide gel electrophoresis (SDS-PAGE). The two left lanes and the two right lanes of each immunoblot were loaded with equal amounts of cytosolic and mitochondrial extracts, respectively. The immunoblots displayed above are representative of at least three biological replicates of EG103 and EG110 yeast cell cultures grown in the absence and presence of aspirin in YPE medium. Cropping of immunoblot images was carried out using Microsoft Word. The full-length untouched immunoblot images are presented in Supplementary Figures S2 and S3.

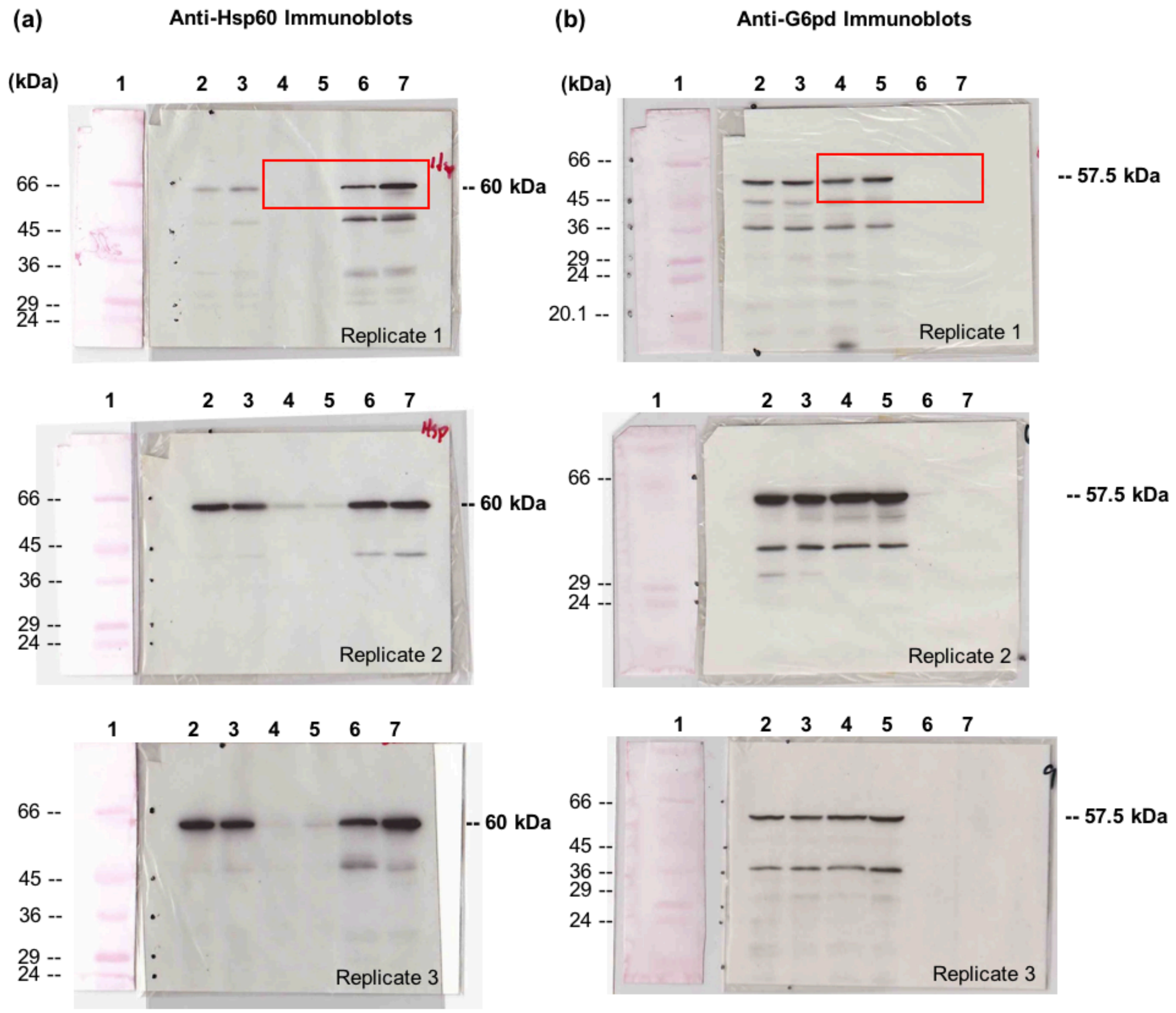

Supplementary Figure S2. Immunoscreening by chemiluminescence to confirm lack of cross-contamination between cytosolic and mitochondrial extracts of *Saccharomyces cerevisiae* EG110 cells: full-length blots. Immunoscreening by chemiluminescence for (a) mitochondrial matrix protein Hsp60 (using monoclonal mouse anti-Hsp60[73], Abcam, ab59458, 1:1000) and (b) cytosolic protein glucose-6-phosphate dehydrogenase (G6pd) (using polyclonal rabbit anti-G6pd[72], Sigma-Aldrich, A9521, 1:7500) was carried out against molecular weight (MW) marker proteins (Sigma, Catalog number: SDS7), in the total cell extract (TCE), cytosolic extract (Cyto) and mitochondrial extract (Mito) of *S. cerevisiae* manganese superoxide-dismutase (MnSOD)-deficient EG110 cells grown aerobically in ethanol medium (YPE) for 48 h in the absence and presence of aspirin (ASA). Lanes 1, MW Marker; Lanes 2, TCE; Lanes 3, TCE + ASA; Lanes 4, Cyto; Lanes 5, Cyto + ASA; Lanes 6, Mito; Lanes 7, Mito + ASA. The electroblotting on to the nitrocellulose membranes was carried out from 5% (w/v) stacking, 12% (w/v) resolving acrylamide gels after sodium dodecyl sulfate polyacrylamide gel electrophoresis (SDS-PAGE). Equal amounts of TCE, cytosolic and mitochondrial extracts were loaded into the wells. The immunoblot images are displayed in full, with their original light exposure. The red rectangle shows the cropped region from the original immunoblot images used for Figure S1. Cropping of representative blots for Supplementary Figure S1 was carried out using Microsoft Word.

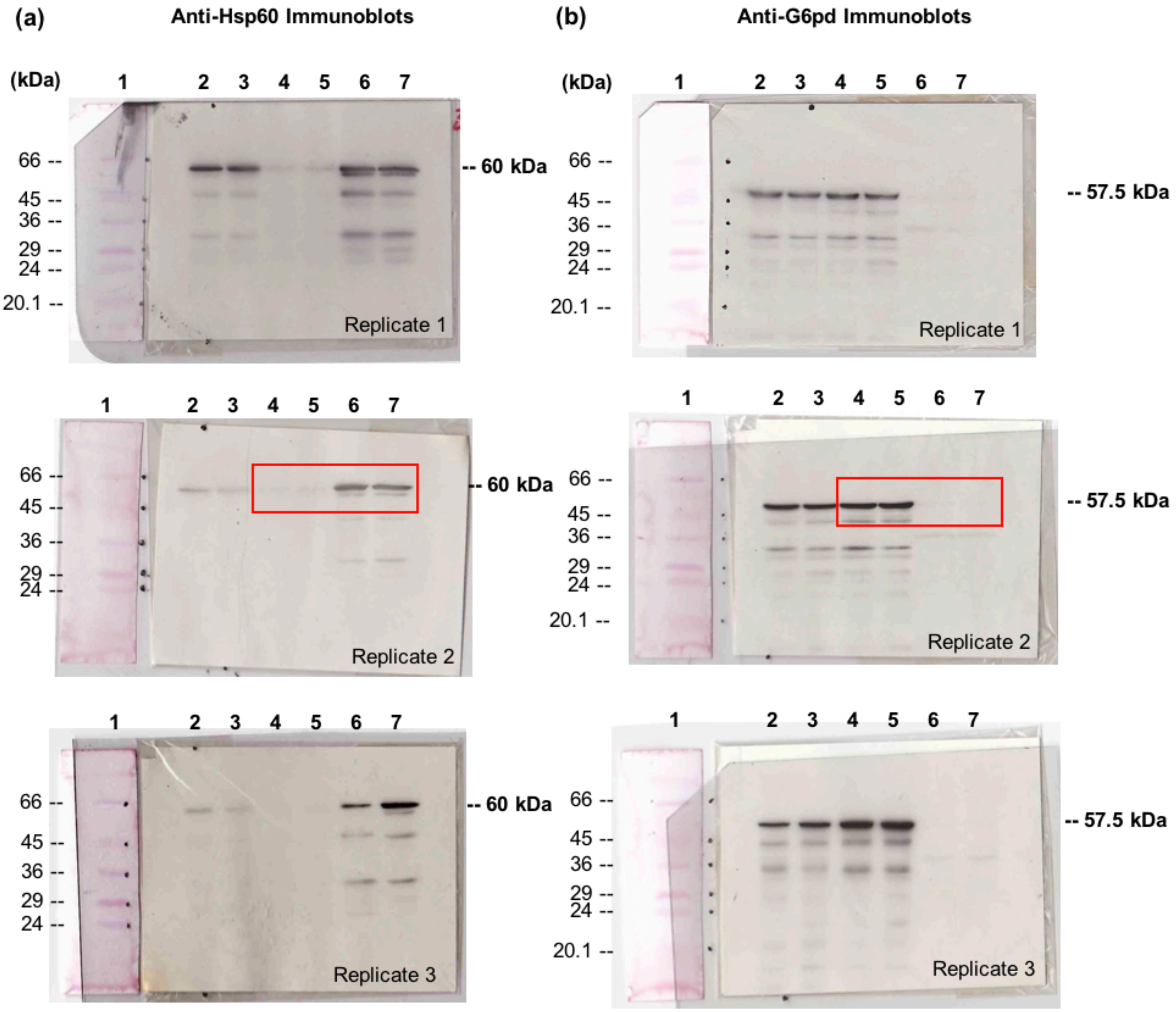

Supplementary Figure S3. Immunoscreening by chemiluminescence to confirm lack of cross-contamination between cytosolic and mitochondrial extracts of *Saccharomyces cerevisiae* EG103 cells: full-length blots. Immunoscreening by chemiluminescence for (a) mitochondrial matrix protein Hsp60 (using monoclonal mouse anti-Hsp60[73], Abcam, ab59458, 1:1000) and (b) cytosolic protein glucose-6-phosphate dehydrogenase (G6pd) (using polyclonal rabbit anti-G6pd[72], Sigma-Aldrich, A9521, 1:7500) was carried out against molecular weight (MW) marker proteins (Sigma, Catalog number: SDS7), in the total cell extract (TCE), cytosolic extract (Cyto) and mitochondrial extract (Mito) of *S. cerevisiae* wild-type EG103 cells grown aerobically in ethanol medium (YPE) for 48 h in the absence and presence of aspirin (ASA). Lanes 1, MW Marker; Lanes 2, TCE; Lanes 3, TCE + ASA; Lanes 4, Cyto; Lanes 5, Cyto + ASA; Lanes 6, Mito; Lanes 7, Mito + ASA. The electroblotting on to the nitrocellulose membranes was carried out from 5% (w/v) stacking, 12% (w/v) resolving acrylamide gels after sodium dodecyl sulfate polyacrylamide gel electrophoresis (SDS-PAGE). Equal amounts of TCE, cytosolic and mitochondrial extracts were loaded in the wells. The immunoblot images are displayed in full, with their original light exposure. The red rectangle shows the cropped region from the original immunoblot images used for Figure S1. Cropping of representative blots for Supplementary Figure S1 was carried out using Microsoft Word.

a)

b)

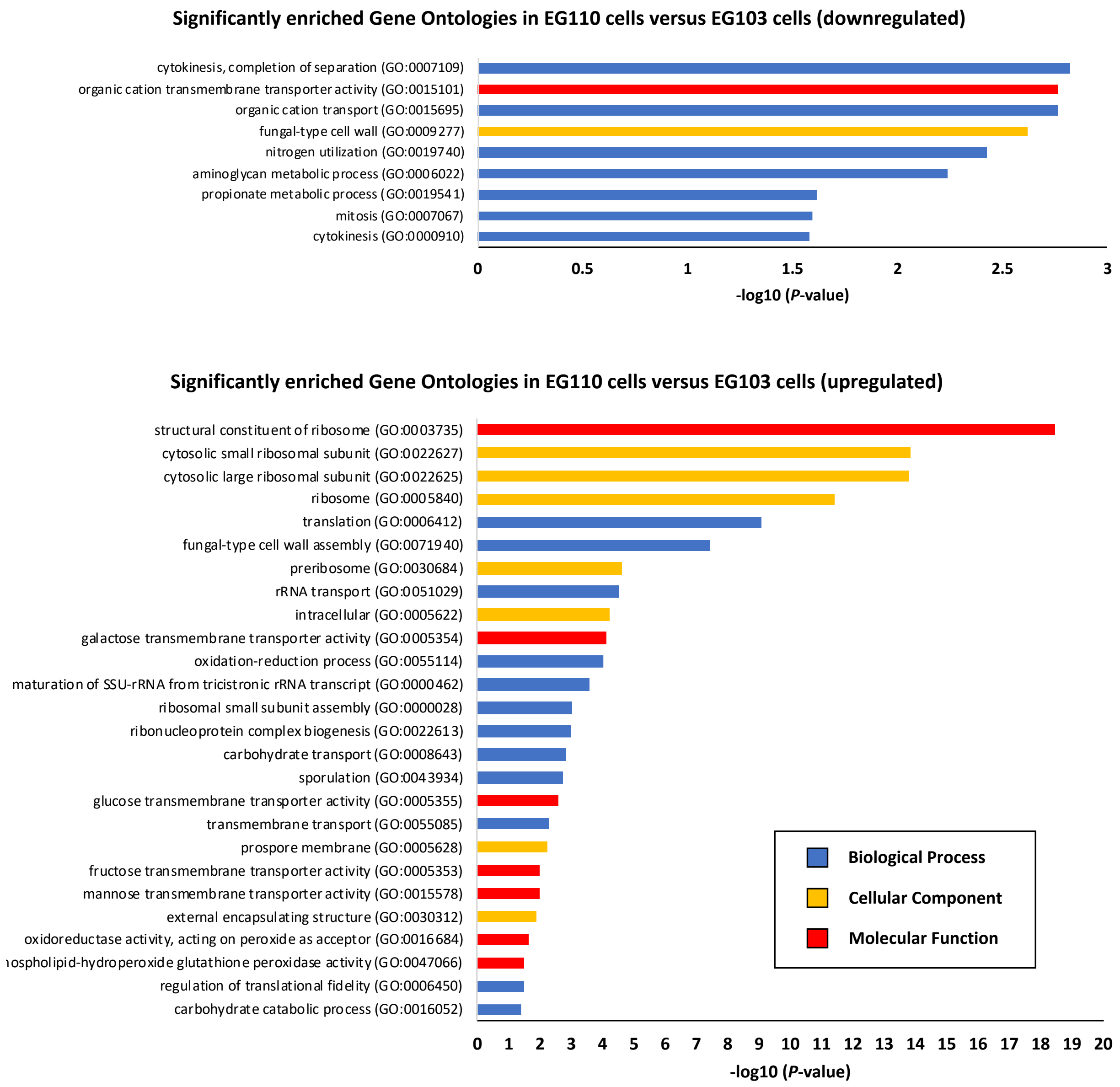

Supplementary Figure S4. Gene Ontology (GO) analysis of differentially expressed mRNA transcripts in aspirin-untreated MnSOD-deficient EG110 yeast cells relative to wild-type EG103 yeast cells. All statistically significant GO terms of (a) downregulated and (b) upregulated mRNA transcripts are shown, each belonging to one of the following GO categories: biological processes (blue bars), cellular components (yellow bars) or molecular function (red bars). The calculated -log10 (*P*-value) values reflect the statistical significance of each GO term enrichment.

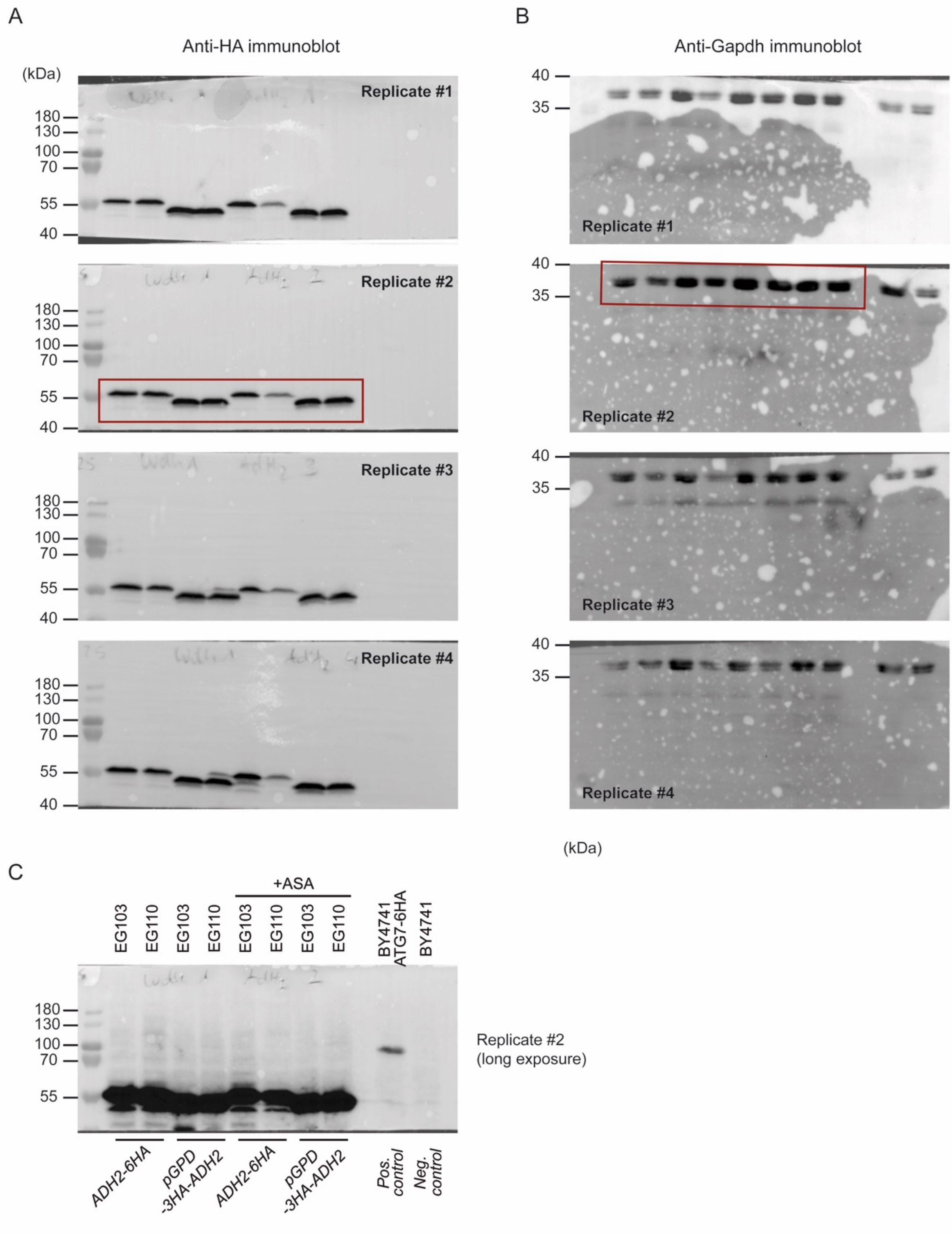

Supplementary Figure S5. Full scan images of the immunoblot presented in Figure 5 and similar replicates. Immunoblots were cut along the 40 kDa marker line and probed with commercially available and characterized antibodies specific for (A) HA (Sigma-Aldrich, H9658) or (B) Gapdh (ThermoFisher, MA5-15738, 1:10.000). The immunoblot images were overlayed with the respective colorimetric photograph to visualize the molecular weight marker PageRuler Prestained Protein Ladder (ThermoFisher, #26616). Respective images were obtained using the ChemiDoc Touch system (Bio-Rad) and Image Lab Software (Bio-Rad) version 5.2.1 with automatically detected optimal exposure times to avoid signal saturation (using the automatic exposure option of Image Lab Software). The red rectangle depicts the cropped region from original immunoblot images used for Figure 5. Linear

adjustments (using Adobe Photoshop CS2) of brightness (+20) and contrast (+10) were applied to the total cropped area. (C) Overexposed immunoblot of replicate #2 demonstrating specificity of the HA-specific antibody using BY4741 wild-type yeast extracts as a negative control (Neg. control), and BY4741 *ATG7-6HA* strain[74] extracts as a positive control (Pos. control).

References for Supplementary Figures

72. Guerriero, C.J., Weiberth, K.F. & Brodsky, J.L. Hsp70 targets a cytoplasmic quality control substrate to the San1p ubiquitin ligase. *J. Biol. Chem*. **288**,18506-18520. https://doi.org/10.1074/jbc.M113.475905 (2013).

73. Bivi, N. *et al*. Transcriptome and proteome analysis of osteocytes treated with nitrogen-containing bisphosphonates. *J. Proteome Res*. **8**, 1131-1142. https://doi.org/10.1021/pr8005606 (2009).

74. Eisenberg, T. *et al*. Nucleocytosolic depletion of the energy metabolite acetyl-coenzyme a stimulates autophagy and prolongs lifespan. *Cell Metab*. Mar 4;19(3):431-44. https://doi.org/10.1016/j.cmet.2014.02.010. (2014).
